# Hospital Architecture

**Published:** 1911-02-25

**Authors:** 


### INSTITUTIONAL LETTER-BOX.

### HOSPITAL ARCHITECTURE.

*To the Editor of* THE HOSPITAL.

SIR,—In your issue of February 11, under the heading of "Hospital Architecture," the following sentence is used: "The floor analysed is Doloment." We wish to point out to you that this is an error, as our composition is "Linolite" and in no way connected with "Doloment," which composition is the make of another jointless flooring firm. At the same time, we should like to add that we are always quite willing to supply our esteemed clients with a guarantee.

Kindly see that this correction is inserted in your paper in order to avoid any misunderstanding by your readers.—Yours, faithfully,

THE LINOLITE FLOORING CO., LTD.

Acme Works, Crowe's Market,
Seven Sisters Road, N.
February 15.

[Our statement stands. Two composite floors were referred to in the original article, published in our issue of January 28. The one analysed was "Doloment"; the other, which was in use in a grand staircase at the Brussels International Exhibition, was made by the Linolite Flooring Company, Ltd.—ED. THE HOSPITAL.]

